# METTL1 interacts with XPO5 to modulate pre-miRNA export

**DOI:** 10.1093/nar/gkag037

**Published:** 2026-01-27

**Authors:** Zhongwen Cao, Xingyuan Chen, Yuxiang Sun, Yinsheng Wang

**Affiliations:** Environmental Toxicology Graduate Program, University of California, Riverside, CA 92502, United States; Environmental Toxicology Graduate Program, University of California, Riverside, CA 92502, United States; Environmental Toxicology Graduate Program, University of California, Riverside, CA 92502, United States; Department of Chemistry, University of California, Riverside, CA 92502, United States; Environmental Toxicology Graduate Program, University of California, Riverside, CA 92502, United States; Department of Chemistry, University of California, Riverside, CA 92502, United States

## Abstract

While METTL1 is a well-established m^7^G writer protein, its protein–protein interaction network remains largely unexplored. To map the METTL1 interactome in HEK293T cells, we employed APEX2-mediated proximity labeling coupled with LC-MS/MS analysis. This approach allowed for the identification of 60 and 18 unique proteins significantly enriched in the METTL1 proximity proteome compared to enhanced green fluorescent protein (EGFP) and nuclear localization signal (NLS) controls, respectively. Among these proteins, we found exportin-5 (XPO5), a nuclear export factor critical for pre-miRNA transport. We validated the METTL1–XPO5 interaction by co-immunoprecipitation and western blot analysis. Strikingly, genetic ablation of METTL1 caused XPO5 to redistribute to the cytosol, which in turn accelerated pre-miRNA export and enhanced miRNA maturation. This function of METTL1 was independent of its canonical m^7^G methyltransferase activity. Mechanistically, we found that METTL1 facilitates ERK-mediated phosphorylation of XPO5, thereby promoting its nuclear retention. Accordingly, constitutive activation of ERK was sufficient to restore nuclear XPO5 localization in METTL1-deficient cells. In summary, our study uncovers a non-canonical role for METTL1 in regulating the subcellular distribution of XPO5 and pre-miRNA export, revealing a novel mechanism of miRNA maturation that extends METTL1’s function beyond m^7^G methylation.

## Graphical Abstract

**Graphical Abstract:**
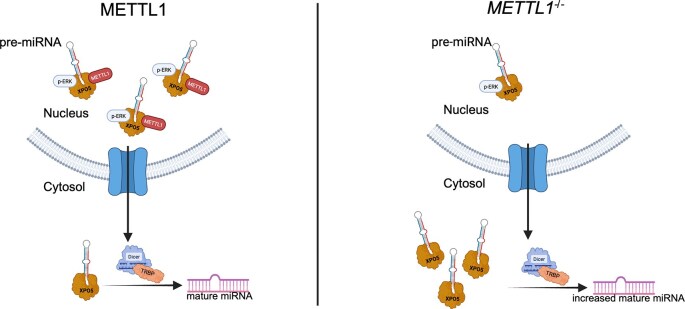

## Introduction

Epitranscriptomic modifications represent a crucial layer of post-transcriptional gene regulation, influencing messenger RNA (mRNA) structure, stability, splicing, and translation [1, 2]. Among these, *N*7-methylguanosine (m^7^G) is a ubiquitous modification found in tRNA, miRNA, and eukaryotic mRNA and has emerged as a key regulator of cellular processes [3–7]. METTL1, a prominent m^7^G “writer,” is frequently dysregulated in various cancers, underscoring its functional importance [7, 8]. Concurrently, the development of advanced sequencing techniques such as m^7^G-RIP-seq has enabled the transcriptome-wide mapping of m^7^G, revealing hundreds of modification sites and further illuminating the role of METTL1 in RNA biology [5, 6, 9].

Beyond its catalytic function, METTL1 may also operate through protein–protein interactions (PPIs). The recent determination of the METTL1/WDR4 complex crystal structures, coupled with evidence that knockdown of either protein drastically reduces m^7^G levels, highlights the functional significance of the METTL1 interactome [10, 11]. This concept is supported by broader evidence that RNA-modifying enzymes can possess non-catalytic, “moonlighting” activities. For example, the *Escherichia coli* 5-methyluridine methyltransferase TrmA acts as a tRNA chaperone independently of its enzymatic function [12]. However, aside from its obligate partner WDR4, the METTL1 interactome remains largely unexplored, and it represents a significant knowledge gap.

To address this, we employed an APEX2-based proximity labeling approach [13], in combination with liquid chromatography-tandem mass spectrometry (LC-MS/MS) analysis, to map the METTL1 interactome. This method offers high labeling efficiency, rapid labeling kinetics, and high spatial resolution, making it particularly powerful for capturing transient and/or weak PPIs [13–15]. Furthermore, when coupled with affinity pull-down of biotinylated peptides, APEX2 allows for the identification of biotinylated peptides and biotinylation sites, increasing specificity and providing potential insights into interaction domains [16]. Using this strategy, we identified exportin-5 (XPO5) as a novel METTL1-interacting protein. We further demonstrate a previously unrecognized, catalysis-independent function for METTL1 in regulating miRNA maturation by modulating XPO5’s subcellular localization. This discovery expands the functional repertoire of METTL1 beyond its canonical m^7^G methylation role.

## Materials and methods

### Cell culture

HEK293T and the isogenic *METTL1*^−/−^ cells were cultured in Dulbecco’s modified Eagle medium (DMEM) containing 10% fetal bovine serum (Invitrogen) and 1% penicillin/streptomycin at 10 000 U/ml (Thermo Fisher). The cells were cultured at 37°C in a humidified atmosphere containing 5% CO_2_. All experiments were conducted using cells that were within 30 passages. The cells were verified to be free from mycoplasma contamination using the LookOut Mycoplasma PCR Detection Kit (MP0035, Sigma–Aldrich).

### Plasmids

The METTL1-V5-APEX, EGFP-V5-APEX, and APEX2-NLS plasmids were constructed by inserting the coding sequence of full-length METTL1, enhanced green fluorescent protein (EGFP), and nuclear localization signal (NLS) into the NotI and EcoRI sites (New England Biolabs) of the modified Mito-V5-APEX empty vector (Addgene, plasmid #124617) [17]. The Flag-HA-METTL1 plasmid was constructed by substituting the coding sequence of YTHDC1 in Flag-HA-YTHDC1 (Addgene, plasmid # 85167) with the full-length METTL1 sequence, utilizing EcoRI and XhoI (New England Biolabs). Catalytically inactive mutant of Flag-HA-METTL1 was generated from Flag-HA-METTL1 using KLD Enzyme Mix (New England Biolabs). Flag-HA-MEK1 was constructed by replacing the YTHDC1 coding sequence in the Flag-HA-YTHDC1 plasmid with the CDS of MEK1 amplified from a complementary DNA (cDNA) library. Flag-MEKDD was subsequently constructed by introducing the S218D and S222D mutations into the Flag-HA-MEK1 construct. The pRK7-3×Flag empty vector control plasmid was obtained from Addgene (plasmid #8996). The sequences of all the constructed plasmids were confirmed by Sanger sequencing.

### Cell lysis and proteomic sample preparation

HEK293T cells at 50% confluency were transfected with METTL1-V5-APEX2, EGFP-V5-APEX2, and APEX2-NLS, respectively, using TransIT-X2 transfection reagent (Mirus Bio). After 24 h, the cells were incubated with 500 μM biotin phenol (Sigma) at 37°C for 30 min. APEX proximity labeling was initiated by incubating with 1 mM hydrogen peroxide for 60 s, and the reaction was terminated using a quencher solution containing Trolox (Sigma), sodium azide (Sigma), and sodium ascorbate (Sigma) in 1× PBS. The treated cells were lysed with cytoplasmic lysis buffer [10 mM Tris–HCl, pH 8.0, 0.34 M sucrose, 3 mM CaCl_2_, 2 mM MgCl_2_, 0.1 mM ethylenediaminetetraacetic acid (EDTA), 1 mM DTT, and 0.5% NP40] containing protease inhibitor cocktail (Sigma–Aldrich). The remaining pellet was further lysed using 8 M urea with protease inhibitor cocktail at room temperature to obtain the nuclear proteome. The supernatants were collected, and the protein concentrations were quantified using Bradford assay (Bio-Rad). Equal amounts of proteins were added to the centrifugal filters with a molecular weight cutoff of 10 kDa (Fisher) and denatured with 8 M urea. The denatured proteins were incubated sequentially with 10 mM dithiothreitol (DTT) and 55 mM iodoacetamide (IAA) at 37°C for 1 h and at room temperature in the dark for 30 min for cysteine reduction and alkylation, respectively. After three times buffer exchange with 50 mM ammonium bicarbonate, the proteins were digested with trypsin (Promega) overnight at an enzyme/protein ratio of 1:100. The peptides were reconstituted in IAP buffer (500 mM MOPS, 100 mM HNa_2_PO_4_, and 500 mM NaCl, pH 8.0), followed by incubation with anti-biotin beads (Immunechem) at room temperature for 2 h. The beads were then washed sequentially twice with IAP buffer and water, respectively. Biotinylated peptides were subsequently eluted from the beads by boiling at 60°C with 100 μl of 0.15% trifluoroacetic acid (TFA) three times. The eluent was desalted using OMIX C18 pipette tips (Agilent Technologies) and dried using a Speed-vac.

### Liquid chromatography-tandem mass spectrometry and data analysis

The desalted peptides were further resuspended in 0.1% formic acid, and half of the samples were subjected to LC-MS/MS analysis on an Orbitrap Fusion Lumos Tribrid mass spectrometer (Thermo Fisher Scientific), which was equipped with a high-field asymmetric-waveform ion mobility spectrometry (FAIMS) and coupled with an EASY-nLC 1200. The peptide samples were loaded onto a custom-packed 3-cm trapping column comprised of 5 μm C18 resin with 0.1% formic acid, and peptides were subsequently eluted onto a 20-cm analytical column packed with 3 μm C18 beads, where the flow rate was 300 nl/min. The mass spectrometer was operated in a data-dependent acquisition mode, where the top 25 most abundant ions detected in MS were chosen for fragmentation by higher-energy collisional activation, and MS/MS were acquired in the Orbitrap at a resolution of 50 000. The raw LC-MS/MS data files were converted into mzXML format with the FAIMS generator software and searched using MaxQuant against the Uniprot database (UP000005640_9606). Cysteine carbamidomethylation was set as a fixed modification, and methionine oxidation, N-terminal acetylation, and biotinylated tyrosine, cysteine, histidine, and tryptophan were set as variable modifications. A maximum of two trypsin missed cleavages were permitted, and the peptides were filtered at 1% false discovery rate (FDR). Label-free quantification was employed with a minimum ratio count of 2. The match between runs option was implemented with a match time window of 0.7 min.

The MS proteomics data were deposited into the ProteomeXchange Consortium via the PRIDE partner repository with the dataset identifier PXD066690.

### Immunoprecipitation and western blot

The cells were lysed in RIPA lysis buffer (50 mM Tris–HCl, pH 7.4, 150 mM NaCl, 2 mM EDTA, 15 mM MgCl_2_, 1% NP-40, 0.5% sodium deoxycholate, 0.1% SDS) containing protease inhibitor cocktail. After lysis on ice for 30 min, the samples were centrifuged at 16 000 × *g* for 30 min, and the supernatant was collected. The protein concentrations were determined by using the Bradford assay, and 1.0 mg of protein was incubated with pre-blocked anti-Flag M2 beads at 4°C overnight. For monitoring the interaction between ectopically expressed Flag-HA-METTL1 and endogenous XPO5, the collected protein lysate was subsequently incubated with anti-Flag beads in CelLytic™ M containing 1 mM MgCl_2_ and 0.15 U/μl benzonase (Sigma) at room temperature for 2 h. The beads were then washed twice with low- (0.1% SDS, 2 mM EDTA, 20 mM Tris–HCl, pH 8.0, 150 mM NaCl, and 1% Triton X-100) and high-salt buffers (0.1% SDS, 2 mM EDTA, 20 mM Tris–HCl, pH 8.0, 500 mM NaCl, and 1% Triton X-100) and once with LiCl buffer (0.25 M, 1% NP-40, 1% sodium deoxycholate, 1 mM EDTA and 10 mM Tris–HCl, pH 8.0). The proteins were subsequently eluted from the beads in a 2× sodium dodecyl sulfate–polyacrylamide gel electrophoresis (SDS–PAGE) sample loading buffer and subjected to western blot analysis.

Antibodies recognizing human XPO5 (Proteintech, #28628-1-AP; 1:2000), V5 (Proteintech, #14440-1-AP; 1:2000), streptavidin (Thermo Scientific, #S911), METTL1 (Proteintech, #14994-1-AP; 1:1000), ERK (Santa Cruz, SC-514302; 1:100), and p-ERK (Cell Signaling, #4370T; 1:1000) were used as primary antibodies for western blot analysis. Goat anti-rabbit IgG (whole molecule)-peroxidase antibody (Sigma, #A0545) and m-IgGκ BP-HRP (Santa Cruz Biotechnology, #sc-516102) were employed as secondary antibodies. Anti-GAPDH (Santa Cruz Biotechnology, #sc-32233, 1:5000), Anti-Lamin B1 (Proteintech, #66095-1-Ig; 1:5000), and Anti-α-tubulin (Santa Cruz, sc-32293, 1:5000) were used for internal control to confirm equal protein loading.

### Real-time quantitative PCR analysis of pre-miRNA and mature miRNA levels

Pre-miRNAs were extracted according to previously published procedures [17]. HEK293T cells and the isogenic METTL1-knockout cells were plated in six-well plates at 50% confluence, and the cytosolic fraction was isolated 24 h post-seeding using Cytoplasmic Extraction Reagents I and II. The nuclear fraction was extracted from the nuclear pellet using 50 μl of Nuclear Extraction Reagent (NER) (Thermo Scientific, #78833). RNA was then isolated from the cytosolic and nuclear fractions using TRIzol treatment, followed by cDNA synthesis using a previously established method [17]. Approximately 2 μg of RNA was denatured and annealed by incubating with reverse primers containing random hexamers specific to the four mature miRNAs at 70°C for 5 min. The RNA was then rapidly chilled on ice, followed by reverse transcription at 42°C for 1 h and 75°C for 15 min using 100 units of M-MLV reverse transcriptase (Promega), M-MLV buffer, dNTPs, and an RNase inhibitor. Pre-miRNA primers and Luna^®^ Universal qPCR Master Mix were subsequently added, and real-time quantitative polymerase chain reaction (RT-qPCR) was conducted using a Bio-Rad iCycler system under the following conditions: 95°C for 3 min, followed by 55 cycles at 95°C for 15 s, 55°C for 30 s, and 72°C for 45 s. Primer sequences are listed in Supplementary Table S1. The levels of pre-miRNA were normalized to that of *GAPDH* mRNA.

Mature miRNAs were isolated as described previously [17]. Cells were seeded in six-well plates at 50% confluence, and total RNA was extracted 24 h post-transfection using TRIzol, followed by cDNA synthesis. Approximately 1 μg of RNA was reverse-transcribed using 100 units of M-MLV reverse transcriptase (Promega), 1 unit of poly(A) polymerase (New England Biolabs), and a 5′-tagged (CAGGTCCAG) oligo(dT)_15_ primer. The reaction was continued at 42°C for 1 h, and the reverse transcriptase was inactivated by heating at 95°C for 5 min. RT-qPCR was performed using Luna^®^ Universal qPCR Master Mix on a Bio-Rad iCycler system. Primer sequences are listed in Supplementary Table S1. The levels of mature miRNAs were normalized to that of *GAPDH* mRNA.

Rescue experiments using wild-type (WT) METTL1 and its catalytically inactive counterpart (LFPD to AFPA mutant, METTL1^AFPA^) were conducted to quantify both pre-miRNA and mature miRNA levels of the four miRNAs [4]. METTL1-knockout cells were seeded in six-well plates at 35% confluence and transfected the following day with plasmids encoding WT and catalytically inactive METTL1. RNA extraction and RT-qPCR for pre-miRNA and mature miRNA were performed as described earlier.

## Results

### APEX2 proximity labeling followed by affinity pull-down of biotinylated peptides for assessing the interactome of METTL1

**Figure 1.**
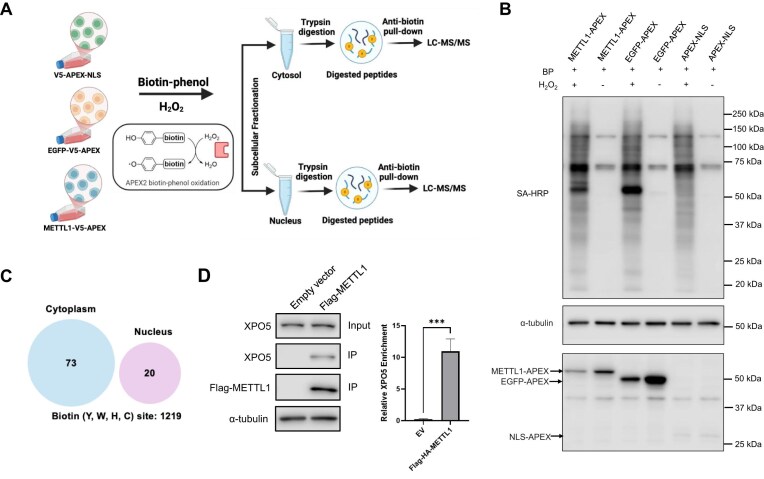
APEX-based proximity labeling led to the identification of XPO5 as an interaction partner of METTL1. (**A**) A schematic illustration of the APEX labeling workflow. (**B**) Western blot analysis showing comparable APEX2 labeling efficiencies among METTL1-APEX, EGFP-APEX, and APEX-NLS. HEK293T cells were transfected with the respective plasmids for 24 h, treated with biotin phenol for 30 min, and subsequently exposed to H_2_O_2_ for 1 min or left untreated. Biotinylated proteins were detected using streptavidin-horseradish peroxidase (SA-HRP). α-tubulin was used as a control to confirm equal protein loading. (**C**) Venn diagrams of proteins identified in the METTL1 proximal proteome. (**D**) Co-IP followed by western blot analysis in HEK293T cells showed a preferential interaction between overexpressed Flag-tagged METTL1 and endogenous XPO5. HEK293T cells were transfected with empty vector or Flag-tagged METTL1, followed by anti-Flag pull-down and western blot analysis. α-tubulin served as a loading control. XPO5 enrichment was quantified by normalizing the amount of immunoprecipitated XPO5 to its corresponding input level (mean ± SEM, *** *P *< .0001, unpaired *t*-test, *n* = 3).

In this study, we set out to apply the APEX-based proximity labeling in conjunction with LC-MS/MS analysis to investigate the interaction proteomes of METTL1 (Fig. 1A). In this vein, recent X-ray crystal structure studies revealed the importance of the N-terminal domain of METTL1 in interacting with WDR4 and orchestrating catalysis [10, 11]; thus, we strategically placed the V5-APEX tag on the C-terminus of METTL1 so as not to disrupt its catalytic activity. To attain a comprehensive identification of METTL1-interacting proteins, we systematically titrated the quantity of METTL1-APEX plasmid required (Supplementary Fig. S1A) and integrated subcellular fractionation with our APEX-based workflow. In this context, APEX2-V5-EGFP and APEX2-V5-NLS serve as the controls for the cytoplasmic and nuclear fractions, respectively, allowing us to distinguish true METTL1-proximal interactions from compartment-specific background labeling. Because overexpression and fusion tags can potentially perturb protein behavior, we optimized the amount of the METTL1-APEX plasmid used (Supplementary Fig. S1A) to minimize nonspecific labeling and verified that METTL1-APEX2 is localized to both the cytosolic and nuclear compartments, in a pattern consistent with endogenous METTL1 (Supplementary Fig. S1B). We confirmed the similar labeling efficiencies of the three APEX plasmids by western blot following exposure to biotin-phenol and hydrogen peroxide (Fig. 1B). After APEX labeling, we isolated the cytoplasmic and nuclear protein fractions, digested them with trypsin, enriched biotinylated peptides from the ensuing digestion mixture using anti-biotin beads, and subjected them to LC-MS/MS analysis (Fig. 1A).

Using a threshold cutoff of at least 1.5-fold, we were able to identify 73 and 20 biotinylated peptides, derived from 60 and 18 unique proteins, that were selectively enriched in the proximity proteomes of METTL1, out of a total of 1219 biotinylated peptides, in the cytoplasmic and nuclear fractions, respectively (Fig. 1C). The STRING analysis highlights RNA-binding proteins and nuclear factors, including members of the hnRNP family, splicing regulators (e.g. U2AF2), and chromatin-associated proteins, suggesting that METTL1 is closely associated with nuclear RNA processing and regulatory complexes (Supplementary Fig. S2A). Notably, the greater number of proteins that appeared in the cytoplasmic fraction in our MS data is in keeping with the subcellular distribution of endogenous METTL1 in HEK293T cells, as noted above. To understand and elucidate the biological processes of the identified proximal proteins of METTL1, we decided to focus on the proteins in the cytosol by performing Gene Ontology (GO) analysis, and the results showed the perturbations in RNA binding, nucleic acid binding, etc. (Supplementary Fig. S1B).

### APEX proximity labeling uncovered an interaction of METTL1 with XPO5

After combining the results obtained from all three biological replicates, our affinity enrichment of biotinylated peptides in conjunction with LC-MS/MS analysis led to the identification of exportin-5 (XPO5) as one of the most enriched proteins in the cytoplasmic proximity proteome of METTL1 over the EGFP control (Supplementary Table S2). We first manually examined the quantification results based on a unique peptide of XPO5, specifically, AGGFVVGY*TSSGNPIFR (Y* represents biotinylated tyrosine). We found that the peak areas observed in the selected-ion chromatograms for the [M + 3H]^3+^ ion of the peptide obtained from the proximity proteome samples of METTL1-APEX were markedly larger than those of EGFP-APEX (Supplementary Fig. S3A). While the stronger association of XPO5 with METTL1 compared to EGFP control is not statistically significant (Supplementary Fig. S3A), the subsequent validation confirmed the interaction of XPO5 with METTL1 (*vide infra*). The sequence of the XPO5 peptide was confirmed by the MS/MS analysis of the [M + 3H]^3+^ ion (Supplementary Fig. S3B). The results from AlphaFold-based structural predictions indicate that XPO5 is capable of interacting directly with METTL1, revealing putative binding interfaces that may underlie this interaction (Supplementary Fig. S3C). These models suggest a physical basis for the observed interactions between METTL1 and XPO5.

XPO5, in conjugation with RAN-GTP, plays an important role in the nuclear-cytoplasmic transport of precursor-miRNAs (pre-miRNAs) [18–20]. MicroRNAs, which are a type of ~22-nucleotide-long non-coding small RNAs, are transcribed from DNA to form primary miRNA and subsequently processed into precursor miRNA by Drosha and DGCR8 within the nucleus [21]. To become mature miRNA, pre-miRNA needs to be transported to the cytoplasm via XPO5/RAN-GTP, followed by Dicer cleavage. Mature miRNA is capable of regulating mRNA levels through annealing with, inducing degradation, and suppressing translation of specific target mRNA [22]. METTL1 was previously shown to install m^7^G on miRNAs, e.g. miR-let-7 [6]. Our identification of XPO5 in the proximity proteome of METTL1 led us to hypothesize that METTL1 may also regulate the miRNA pathway by modulating nuclear-cytoplasmic transport of pre-miRNA.

To test the above hypothesis, we first validated the interaction of METTL1 and XPO5 using co-immunoprecipitation (Co-IP) and western blot analysis. Our results confirm the interaction of METTL1 with XPO5, and this interaction occurs independently of DNA or RNA, as our Co-IP experiment was conducted in the presence of benzonase (Fig. 1D).

### Genetic ablation of METTL1 induces the redistribution of pre-miRNA and alters the level of mature miRNA

**Figure 2.**
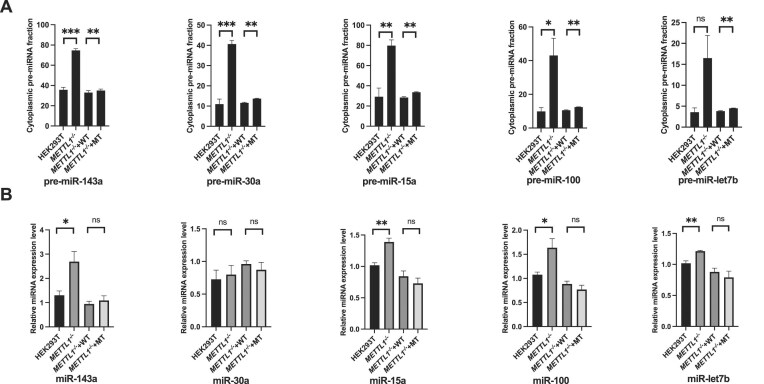
METTL1 modulates the nucleocytoplasmic transport of pre-miRNAs through a mechanism that is independent of its catalytic activity. (**A, B**) RT-qPCR analysis of pre-miRNAs (top) and the corresponding cytoplasmic miRNAs (bottom) in the HEK293T cell lines. “*METTL1*^−/−^” indicates HEK293T cells with METTL1 being knocked out; “*METTL1*^−/−^ + WT” and “*METTL1*^−/−^ + MT” represent *METTL1*^−/−^ cells reconstituted with WT METTL1 and its catalytically inactive mutant, respectively. All data were normalized to the mRNA level of *GAPDH*. The *P*-values were calculated using an unpaired, two-tailed Student’s *t*-test: ns, not significant (*P *> .05); *, .01 ≤ *P* < .05; **, .001 ≤ *P* < .01; ***, *P* < .001.

miRNAs are essential single-stranded non-coding RNAs that play critical roles in RNA silencing and post-transcriptional regulation of gene expression [22]. A well-established function of XPO5 is to mediate the nuclear export of pre-miRNAs, which are subsequently processed by Dicer into mature miRNA in the cytoplasm [18–20]. Given the central role of XPO5 in pre-miRNA transport, we investigated whether METTL1 affects the nucleocytoplasmic transport of pre-miRNAs through its interaction with XPO5. To this end, we employed CRISPR–Cas9 gene editing to ablate METTL1 in HEK293T cells, and successful ablation of the gene was confirmed by western blot analysis (Supplementary Table S3 and  Supplementary Fig. S1B). We then selected a panel of representative miRNAs known to be highly expressed in both humans and mice, including miR-143a, miR-30a, miR-15a, miR-100, and miR-let7b, and examined their levels in parental and *METTL1*^−/−^ cells by employing RT-qPCR analysis [17]. We first examined the distribution of the corresponding pre-miRNAs by performing subcellular fractionation followed by RT-qPCR to quantify their abundance in the cytoplasmic and nuclear compartments. Compared to parental HEK293T cells, *METTL1*^−/−^ cells exhibited a pronounced increase in pre-miRNAs in the cytosol (Fig. 2A), indicating enhanced nuclear export of precursor miRNAs.

As pre-miRNAs must be transported to the cytoplasm for Dicer-mediated processing, we next assessed the abundance of mature miRNAs, and our results revealed that the mature miRNA levels for all tested candidates were significantly increased in *METTL1*^−/−^ cells relative to control cells, suggesting that METTL1, through its interaction with XPO5, influences miRNA biogenesis by modulating nucleocytoplasmic transport of pre-miRNAs (Fig. 2B). The lack of change for miR-30a may arise from limited Dicer processing due to substrate stability and selectivity [23, 24] or from terminal-loop masking by RNA-binding proteins [25].

We also investigated whether the regulatory effect of METTL1 on pre-miRNA export entails its enzymatic activity. To address this, we generated a catalytically inactive mutant METTL1 by altering the active site residues from LFPD to AFPA (METTL1^AFPA^), which disrupts its m^7^G methyltransferase activity [4]. We performed rescue experiments in METTL1 knockout cells by reintroducing either METTL1^WT^ or METTL1^AFPA^, where we confirmed, by western blot analysis, the similar levels of expression of METTL1^WT^ and METTL1^AFPA^ (Supplementary Fig. S4A). Our results from RT-qPCR analysis revealed that reconstitution of *METTL1*^−/−^ cells with METTL1^WT^ or METTL1^AFPA^ was able to restore pre-miRNA and mature miRNA levels, indicating that METTL1’s catalytic activity is dispensable for its function in modulating pre-miRNA export (Fig. 2A and B). Together, these data highlight a previously unrecognized, catalysis-independent role of METTL1 in regulating miRNA biogenesis.

### METTL1–XPO5 interaction regulates XPO5’s subcellular distribution

**Figure 3.**
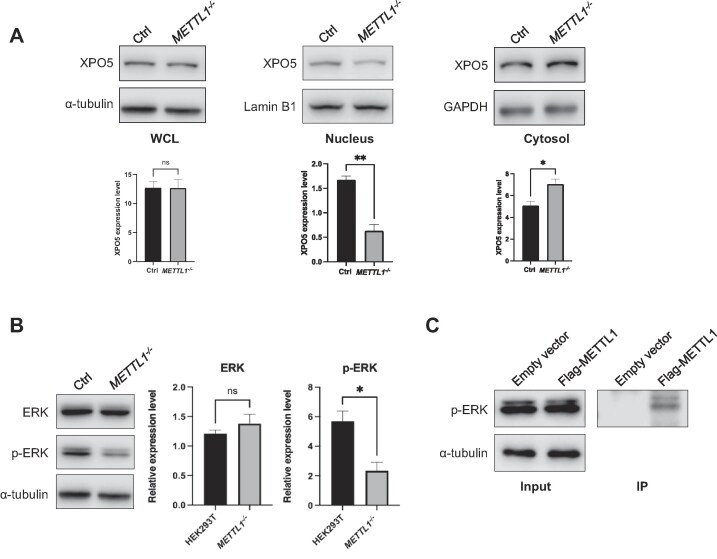
METTL1 promotes nuclear retention of XPO5 through promoting ERK activation. (**A**) Western blot illustrating the XPO5 expression levels in the whole-cell lysates (WCL), as well as the cytoplasmic and nuclear fractions of control and METTL1 knockout cells. The result demonstrates a significant increase in cytosolic XPO5 and a significant decrease in nuclear XPO5 in *METTL1^−^*^/−^ cells, whereas total XPO5 remains unchanged. α-tubulin and GAPDH were used as loading controls for cytosolic fraction, and Lamin B1 as a loading control for the nuclear fraction. (**B**) Western blot analysis of ERK and p-ERK in HEK293T and *METTL1^−^*^/−^ cells. While total ERK expression remained unchanged, p-ERK levels were significantly reduced in the absence of METTL1. (**C**) Co-IP followed by western blot analysis showing the interaction between METTL1 and p-ERK. The *P* values were calculated using paired, two-tailed Student’s *t*-test: ns, not significant (*P* > .05); *, .01 ≤ *P* < .05; **, .001 ≤ *P* < .01.

On the grounds of the previous observation that XPO5’s function in regulating pre-miRNA transport is modulated by its subcellular distribution [26], we examined how loss of METTL1 influences the subcellular distributions of XPO5. Our western blot results revealed no significant difference in the overall expression level of XPO5 between HEK293T and the isogenic *METTL1*^−/−^ cells (Fig. 3A). We, however, observed a significant diminution in the level of XPO5 in the nuclei in *METTL1*^−/−^ cells, which is accompanied by an augmented presence of XPO5 in the cytoplasm (Fig. 3A). These subcellular fractionation data strongly support our hypothesis that the interaction between METTL1 and XPO5 plays a critical role in regulating pre-miRNA export. Specifically, METTL1 ablation resulted in increased cytoplasmic localization of XPO5, which in turn increases the export of pre-miRNAs from the nucleus. This elevated export likely contributes to the observed accumulation of pre-miRNAs in the cytoplasm, thereby giving rise to increased levels of mature miRNAs.

We next sought to elucidate the mechanism by which METTL1 modulates the subcellular distribution of XPO5. Recent studies showed that the phosphorylation state of XPO5 plays a crucial role in influencing its nuclear retention or export, with ERK-mediated phosphorylation promoting the nuclear localization of XPO5 [26, 27]. To investigate whether the METTL1–XPO5 interaction is involved in ERK-mediated regulation, we first examined the ERK phosphorylation status in control and METTL1 knockout cells. Western blot analysis revealed that while total ERK levels remained unchanged, phosphorylated ERK (p-ERK) was significantly reduced in METTL1 knockout cells (Fig. 3B). Furthermore, the co-IP experiment demonstrated that METTL1 interacts with p-ERK, suggesting that METTL1, through its interaction with both XPO5 and p-ERK, may help stabilize the p-ERK-XPO5 complex (Fig. 3C), as previous studies showed that p-ERK can interact with and phosphorylate XPO5, promoting its nuclear retention [26, 27]. Together, the Co-IP result suggests that METTL1, which interacts with both XPO5 and p-ERK, may act as a scaffold to facilitate the formation and/or stabilization of the p-ERK-XPO5 complex, thereby regulating XPO5’s subcellular localization and pre-miRNA export.

**Figure 4.**
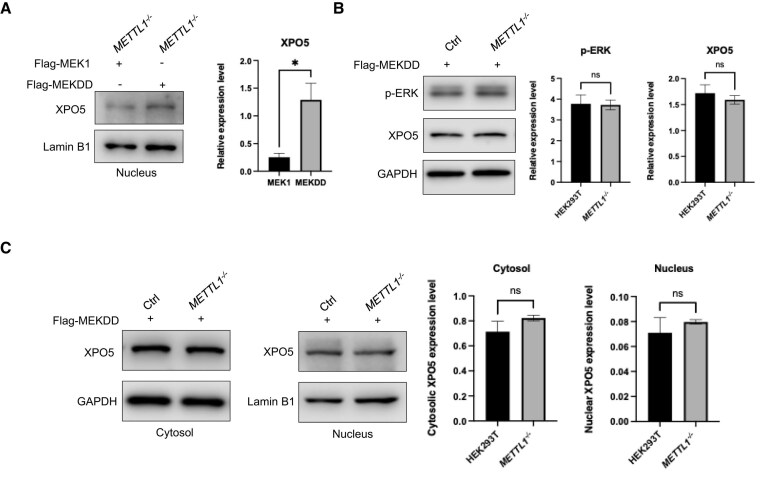
ERK activation restores XPO5’s nuclear retention in *METTL1*^−/−^ cells. (**A**) Western blot analysis of nuclear XPO5 in *METTL1*^−/−^ cells transfected with Flag-MEK1 or Flag-MEKDD. (**B**) Western blot analysis showing p-ERK and XPO5 in HEK293T (labeled as “Ctrl”) and the isogenic *METTL1*^−/−^ cells. GAPDH was used as a loading control. (**C**) Subcellular fractionation analysis of XPO5 in cytosolic and nuclear compartments in HEK293T and *METTL1*^−/−^ cells expressing Flag-MEKDD. GAPDH and Lamin B1 were used as loading controls for the cytosolic and nuclear fractions, respectively. The *P*-values were calculated using a paired, two-tailed Student’s *t*-test: ns, not significant (*P* > .05); *, .01 ≤ *P* < .05.

To test whether ERK activation is sufficient to rescue the altered XPO5 localization observed in METTL1-deficient cells, we ectopically expressed either WT MEK1, which requires upstream signaling to activate the ERK phosphorylation pathway, or a constitutively active variant of MEK1 (Flag-MEKDD), which directly drives ERK phosphorylation, in METTL1 knockout cells [28]. Western blot analysis of nuclear fractions showed that expression of Flag-MEKDD significantly restored the nuclear level of XPO5 compared to Flag-MEK1 (Fig. 4A), indicating that activation of ERK can reverse the loss of nuclear XPO5 elicited by METTL1 depletion. Importantly, while MEKDD overexpression increased p-ERK levels for *METTL1*^−/−^ cells, it did not alter the level of XPO5 in the whole-cell lysate (Fig. 4B), consistent with the previous observation that ERK activity regulates XPO5’s subcellular localization rather than its expression [26].

To further confirm that ERK reactivation rescues the subcellular distribution of XPO5, we performed subcellular fractionation for HEK293T and *METTL1*^−/−^ cells with ectopic overexpression of the constitutively active form of MEK, i.e. Flag-MEKDD. Our results revealed similar subcellular distribution of XPO5 in HEK293T and the isogenic *METTL1*^−/−^ cells with ectopic expression of Flag-MEKDD (Fig. 4C). These results demonstrate that METTL1 promotes the nuclear retention of XPO5, in part, by maintaining ERK activation, and that restoring ERK activity is sufficient to rescue the aberrant subcellular localization of XPO5 in METTL1 knockout cells.

## Discussion

METTL1 is a key RNA methyltransferase responsible for catalyzing the formation of m^7^G, a crucial post-transcriptional RNA modification involved in regulating gene expression and RNA metabolism [4, 5, 7–9, 12]. In complex with its binding partner WDR4, METTL1 catalyzes the installation of m^7^G in specific RNA substrates, particularly tRNAs, thereby improving translation efficiency and fidelity [4, 7, 8, 12]. Previous studies showed that METTL1 installs m^7^G in specific tumor-suppressive miRNAs, e.g. members of the let-7 family [6]. This methylation antagonizes G-quadruplex structures in miRNA precursors, thereby facilitating their processing into miRNA. As a result, METTL1 can influence cell migration and other cellular processes through its effects on miRNA maturation [6]. However, this function of METTL1 in installing m^7^G in miRNAs remains controversial, as different high-throughput mapping approaches yielded conflicting results [6, 29]. In addition, METTL1 can install m^7^G in mRNAs. These m^7^G-harboring transcripts are selectively recognized by Quaking proteins (QKIs), particularly QKI7, which facilitates their localization to stress granules [9]. This mechanism has been shown to sensitize cancer cells to chemotherapy by regulating the translation of stress-responsive mRNAs [9]. Aberrant overexpression of METTL1 has been implicated in various cancers, where it promotes cell proliferation, cell cycle progression, and tumorigenesis [8, 30, 31]. While many studies have focused on the enzymatic activity and oncogenic potential of METTL1, the protein interactome of METTL1 remains largely unknown.

In this study, we adopted APEX2 proximity labeling, coupled with affinity pull-down of biotinylated peptides, to investigate the protein interactomes of METTL1 (Fig. 1A). GO and STRING analysis of the peptides enriched in the proximity proteomes of METTL1 revealed the involvement of several pivotal biological pathways (Supplementary Fig. S2A and B). Among the proteins enriched in the proximity proteome of METTL1, CAPRIN1, an RNA-binding protein implicated in stress granule formation and mRNA transport, was predominantly enriched in the proximity proteome of nuclear METTL1, while U2AF2, a key splicing factor required for early pre-mRNA processing, was enriched in the proximity proteome of cytosolic METTL1 (Supplementary Table S2) [32, 33]. Interestingly, we also identified XPO5 as significantly enriched in the METTL1 cytoplasmic proximal proteome compared to the control group (Supplementary Table S2 and Supplementary Fig. S3). This finding was further validated by co-IP followed by western blot analysis, which revealed a stronger interaction between METTL1 and XPO5 relative to the control group in HEK293T cells (Fig. 1D). Together, these results highlight XPO5 as a key cytoplasmic interactor of METTL1.

XPO5 is known to promote nucleocytoplasmic transport of small endogenous RNAs, including pre-miRNAs, enabling their delivery from the nucleus to the cytoplasm, where Dicer processes them into mature miRNAs [19, 21, 22]. The ensuing mature miRNAs play vital roles in many crucial biological processes, including gene silencing, RNA editing, RNA methylation, and RNA decay [21, 22]. Previous studies suggested that the subcellular distribution of XPO5 itself can influence the efficiency of pre-miRNA export and subsequent maturation [19]. We observed that loss of METTL1 led to increased cytoplasmic localization of several pre-miRNAs and elevated levels of their corresponding mature forms (Fig. 2A and B). Rescue experiments in METTL1-deficient cells showed that both WT and catalytically inactive METTL1 could restore pre-miRNA and mature miRNA levels, suggesting that METTL1 regulates miRNA maturation in a catalysis-independent manner, most likely through its PPI with XPO5 (Fig. 2A and B).

To gain further insights into its mechanism, we examined how METTL1 affects the subcellular distribution of XPO5. Although we observed no significant difference in overall XPO5 protein levels between control and METTL1 knockout cells (Fig. 2A), subcellular fractionation followed by western blot analysis revealed a pronounced shift in XPO5's subcellular localization. In *METTL1^−/−^* cells, nuclear XPO5 levels were significantly reduced, while cytoplasmic XPO5 was markedly increased (Fig. 3A). These findings suggest that METTL1 may play a role in maintaining the proper nuclear localization of XPO5, thereby regulating pre-miRNA export and impacting miRNA maturation.

ERK-mediated phosphorylation of XPO5 was shown to modulate the subcellular distribution of XPO5 through promoting the nuclear retention of XPO5, which impairs the export of pre-miRNAs and leads to reduced levels of mature miRNAs [26, 27]. In light of our findings and these previous observations, we next asked whether ERK activation is affected by METLL1 loss. Notably, genetic ablation of METTL1 led to a pronounced reduction in ERK phosphorylation without altering the overall ERK level (Fig. 3B), suggesting that METTL1 promotes ERK activation. Furthermore, co-IP followed by western blot analysis confirmed an interaction between METTL1 and p-ERK (Fig. 3C). Hence, METTL1 can bind to both XPO5 and p-ERK, which, in conjunction with the previously observed interaction of XPO5 with p-ERK, suggests that METTL1 functions as a scaffold stimulating p-ERK-XPO5 interaction and XPO5 phosphorylation [26].

Consistent with the hypothesis that ERK activity controls XPO5 localization, expression of Flag-MEKDD in *METTL1*^−/−^ cells significantly restored the nuclear localization of XPO5 (Fig. 4A). This rescue occurred in the absence of METTL1, suggesting that ERK activation alone is sufficient to reverse the cytoplasmic accumulation of XPO5 elicited by METTL1 loss. Notably, overexpression of Flag-MEKDD successfully restored p-ERK levels in *METTL1*^−/−^ cells to levels comparable to those in parental HEK293T cells, whereas total XPO5 protein in the whole cell lysates remained the same (Fig. 4B). Subcellular fractionation further confirmed that constitutive ERK activation restored XPO5 distribution in both the cytoplasm and nucleus to levels comparable to those of WT cells (Fig. 4C). Together, these results support a model in which METTL1 maintains XPO5’s nuclear localization through its phosphorylation enabled by activated ERK. Loss of METTL1 led to attenuated interaction between XPO5 and pERK, diminished XPO5 phosphorylation, augmented distribution of XPO5 in the cytosol, and enhanced pre-miRNA export and miRNA maturation. These findings highlight a previously unrecognized mechanism by which METTL1 regulates pre-miRNA trafficking via modulating XPO5-p-ERK interaction, which is independent of METTL1’s catalytic activity.

In conclusion, we uncovered a novel, catalysis-independent role of METTL1 in regulating miRNA biogenesis through PPIs. We identified XPO5 as a METTL1-interacting protein and documented METTL1’s function in modulating XPO5’s subcellular localization through enhancing its phosphorylation by ERK. These findings reveal a role of METTL1 in coordinating protein interactions to modulate pre-miRNA transport, thereby extending its function beyond its canonical role in RNA methylation. Our study, together with the previously published study [6], revealed that METTL1 can regulate miRNA biogenesis not only through Drosha-mediated production of pre-miRNA, but also via XPO5-mediated nucleocytoplasmic transport of pre-miRNA. METTL1 is overexpressed in subsets of human cancers and is known to promote tumorigenesis through its catalytic m^7^G modification of tRNA [8, 30, 31]. Our data suggest a parallel, non-catalytic axis in which METTL1 sustains ERK-dependent nuclear retention of XPO5, thereby restraining pre-miRNA export. We speculate that a resulting reduction of tumor-suppressive miRNAs could cooperate with m^7^G-dependent translational programs to promote tumorigenesis. Moving forward, it will be of interest to investigate other METTL1-interacting proteins identified from our proximity labeling experiment so as to elucidate the broader biological functions of METTL1.

## Supplementary Material

gkag037_Supplemental_File

## Data Availability

The MS proteomics data were deposited into the ProteomeXchange Consortium via the PRIDE partner repository with the dataset identifier PXD066690.
