# Supplementary material for: METTL1 interacts with XPO5 to modulate pre-miRNA export
Source: Nucleic Acids Res. 2026 Jan 27;54(3):gkag037. doi: 10.1093/nar/gkag037 (PMC12839532; doi:10.1093/nar/gkag037)

Supplementary Materials for
"METTL1 Interacts with XPO5 to Modulate Pre-miRNA Export"

Zhongwen Cao[1], Xingyuan Chen[1], Yuxiang Sun[2], and Yinsheng Wang[1,2,*]

[1]Environmental Toxicology Graduate Program, University of California, Riverside, CA 92521, USA.
[2]Department of Chemistry, University of California, Riverside, CA 92521, USA.

[*] To whom correspondence should be addressed. Email: yinsheng@ucr.edu.

Table of Contents:

**Contents**	**Page(s)**
**Table S1**. Primer Sequences used for miRNA quantification with RT-qPCR.	**S2**
**Table S2**. Proteins enriched in the proximity proteome METTL1 based on APEX proximity labeling and LC-MS/MS analysis.	**S3-S5**
**Table S3**. Sequences of sgRNAs for knocking out METTL1.	**S6**
**Figure S1**. Expression optimization and localization of METTL1-APEX for proximity labeling experiments.	**S7**
**Figure S2.** STRING/GO enrichment of proximal proteins of METTL1.	**S8**
**Figure S3.** LC-MS/MS validation and structural mapping of XPO5 peptide enriched by METTL1 proximity labeling. .	**S9**
**Figure S4.** Expression level of wild-type METTL1 and METTL1^{AFPA}	**S10**

Table S1. Primer Sequences used for miRNA quantification in RT-qPCR.

Description	Primer
5′ tag-oligo dT15	5’-CAGGTCCAGTTTTTTTTTTTTTTT -3’
mir-30a Forward	5’-CGCAGTGTAAACATCCTCGACTG -3’
mir-30a Reverse	5’-CAGGTCCAGTTTTTTTTTTTTTTTCTTCCAG -3’
mir-let-7b Forward	5’-GCAGTGAGGTAGTAGGTTGTGT -3’
mir-let-7b Reverse	5’-CAGGTCCAGTTTTTTTTTTTTTTTAACCACAC -3’
mir-15a Forward	5’-CGCAGTAGCAGCACATAATGGTT -3’
mir-15a Reverse	5’-CAGGTCCAGTTTTTTTTTTTTTTTCACAAAC -3’
mir-100 Forward	5’-GCAGAACCCGTAGATCCGAACT -3’
mir-100 Reverse	5’-CAGGTCCAGTTTTTTTTTTTTTTTCACAAGTT -3’
miR-143a Forward	5’-TGCAGTGCTGCATCTCT-3’
miR-143a Reverse	5’-TGCAGTGCTGCATCTCT-3’
Pre-miR-100 Forward	5’-CGTAGATCCGAACTTGTGGT -3’
Pre-miR-100 Reverse	5’-CATACCTATAGATACAAGCTTGT-3’
Pre-miR-15a Forward	5’-TAGCAGCACATAATGGTTTGTG-3’
Pre-miR-15a Forward	5’-TGAGGCAGCACAATATGGCCT-3’
Pre-miR-30a Forward	5’-ATCCTCGACTGGAAGCTGTG -3’
Pre-miR-30a Reverse	5’-CTGCAAACATCCGACTGAAA -3’
Pre-miR-let7a Forward	5’-TGGGATGAGGTAGTAGGTTGTATAG -3’
Pre-miR-let7a Reverse	5’-TATCTCCCAGTGGTGGGTGT -3’
Pre-miR-143 Forward	5’-CTGTCTCCCAGCCTGAGGT -3’
Pre-miR-143 Reverse	5’-CAGACTCCCAACTGACCAGAG -3’
GAPDH Forward	5′-TTCGACAGTCAGCCGCATCTTCTT-3′
GAPDH Reverse	5′-CAGGCGCCCAATACGACCAAATC-3′

Table S2. A list of proteins enriched in the proximity proteome of METTL1 identified from APEX-based proximity labeling, affinity pull-down of biotinylated peptides, and LC-MS/MS analysis. The LFQ intensities of the three replicates from METTL1-APEX were normalized against the three replicates of EGFP-METTL1 and NLS-APEX, respectively. Proteins identified only in the proximity proteome of METTL1 were labeled as M1 only.

Gene names	Peptide ID	METTL1/ EGFP-1	METTL1/ EGFP-2	METTL1/ EGFP-3
BCE1	TC*FLY*LRFFPCLSWMSLK	3.91	3.07	M1 only
TOP3B	FKMTSVC*GH*MTLDFLGK	4.54	4.05	2.72
VAC14	GLEC*SPSTPTMNSYFYKFMINLLK	M1 only	M1 only	3.13
MYO5B	KDVC*SW*STGMQLR	M1 only	M1 only	2.12
RPS10;RPS10P5	DVH*MPKHPELADK	M1 only	M1 only	M1 only
	DVHMPKH*PELADK	M1 only	M1 only	M1 only
PDLIM1	VITNQY*NNPAGLYSSENISNFNNALESK	M1 only	2.45	M1 only
SMAP	INEELESQY*QQSMDSK	M1 only	M1 only	M1 only
HAT1	LLVTDMSDAEQY*R	M1 only	M1 only	M1 only
PPM1G	AY*TGFSSNSER	M1 only	M1 only	3.04
IPO8	TALQEVY*TLAEHR	3.76	3.55	3.14
MTA2	GHLSRPEAQSLSPY*TTSANR	M1 only	M1 only	2.56
SSB	SPSKPLPEVTDEY*K	M1 only	M1 only	M1 only
HMGB1;HMGB1P1;HMGB2	MSSY*AFFVQTCR	M1 only	M1 only	M1 only
HNRNPA1	NQGGY*GGSSSSSSYGSGR	M1 only	13.0	2.21
	NQGGYGGSSSSSSY*GSGR	M1 only	M1 only	2.19
HNRNPA1;HNRNPA1L2	SSGPY*GGGGQY*FAKPR	M1 only	M1 only	2.76
XRCC6	SGWESY*YK	M1 only	M1 only	M1 only
	SGWESYY*K	M1 only	5.64	M1 only
NCL	GQNQDY*R	M1 only	M1 only	M1 only
	SISLY*YTGEK	M1 only	8.32	M1 only
	SISLYY*TGEK	M1 only	8.32	M1 only
HNRNPA2B1	GGSDGY*GSGR	M1 only	M1 only	4.36
TCEA1	DTY*VSSFPR	M1 only	M1 only	M1 only
POLR2A	Y*SPTSPTYPTSPVYTPTSPK	M1 only	M1 only	M1 only
U2AF2	RPHDYQPLPGMSENPSVY*VPGVVSTVVPDSAHK	M1 only	M1 only	M1 only
ARNT	FSEIY*HNINADQSK	M1 only	6.31	2.43
RPA1	VVPIASLTPY*QSK	M1 only	4.57	1.89
HNRNPH1;HNRNPH2	DLNY*CFSGMSDHR	M1 only	M1 only	3.34

MCM4	LASALAPSIY*EHEDIKK	M1 only	M1 only	1.82
FUS	APKPDGPGGGPGGSHMGGNY*GDDR	M1 only	4.92	3.58
EIF4A3	LDY*GQHVVAGTPGR	M1 only	M1 only	1.67
NASP	EAEGSSAEY*K	M1 only	M1 only	2.47
	EQVY*DAMGEKEEAK	5.59	M1 only	2.89
HDGF	ASGY*QSSQK	9.19	5.40	2.91
HNRNPM	FEPY*ANPTK	M1 only	M1 only	M1 only
PSME3	SSNAETLY*	M1 only	3.02	1.65
HNRNPK	RDY*DDMSPR	M1 only	M1 only	6.68
	GGDLMAY*DR	M1 only	M1 only	M1 only
CNBP	DCDLQEDACY*NCGR	M1 only	2.18	M1 only
HIST1H4A	DAVTY*TEHAK	9.42	3.77	2.27
HMGN5	QEAVVEEDY*NENAK	M1 only	M1 only	1.54
ERH	ADTQTYQPY*NK	9.94	6.91	4.40
HNRNPU	GY*FEYIEENK	M1 only	M1 only	M1 only
	PWSQHYHQGYY*	M1 only	M1 only	M1 only
FKBP4	GEGY*AKPNEGAIVEVALEGYYK	2.88	2.27	M1 only
ILF3	AY*AALAALEK	M1 only	4.85	7.06
TRIM28	DHQY*QFLEDAVR	M1 only	M1 only	M1 only
	LSPPY*SSPQEFAQDVGR	M1 only	M1 only	M1 only
MCM6	VSGVDGY*ETEGIR	M1 only	11.10	3.62
PDCD11	AGTY*FSNQAVRACILCVHPR	M1 only	M1 only	M1 only
SART3	AVAAATY*K	M1 only	M1 only	M1 only
EIF4H	ADFDTY*DDR	M1 only	M1 only	M1 only
CPSF6	GPPPTDPYGRPPPY*DR	M1 only	M1 only	M1 only
COPS6	IIIDKEY*YY*TK	M1 only	M1 only	M1 only
C1orf52	SNY*VPPPETYTTEK	M1 only	M1 only	M1 only
PRPF31	VGY*ELKDEIER	M1 only	M1 only	M1 only
HDAC2	AY*SQGGGK	M1 only	M1 only	1.92
KHSRP;FUBP1	AWEEYY*K	M1 only	6.56	3.84
KHSRP	IGQQPQQPGAPPQQDY*TK	M1 only	5.77	3.00
FUBP1	GTPQQIDY*AR	7.05	5.65	3.97
	ITGDPY*K	M1 only	M1 only	12.0
KIAA1143	NQVSY*VRPAEPAFLAR	M1 only	M1 only	2.85
CMBL	ANEAY*PCPCDIGHR	M1 only	M1 only	M1 only
TCEAL4	LY*SENEGMASNQGK	M1 only	M1 only	1.53
HNRNPAB	EVYQQQQY*GSGGR	M1 only	7.69	2.81
MCMBP	SY*EDDDDMDLQPNK	M1 only	M1 only	M1 only

HNRNPUL1	NPPGASTY*NK	M1 only	M1 only	M1 only
XPO5	AGGFVVGY*TSSGNPIFR	M1 only	9.41	M1 only
RBM12	LQNFSY*DQR	M1 only	4.56	2.62
THUMPD1	AQY*VLAK	M1 only	M1 only	M1 only
METTL1	HLPNFFY*K	M1 only	M1 only	M1 only
	VSDY*VQDR	M1 only	M1 only	M1 only
ACIN1	STLADY*SAQK	M1 only	M1 only	M1 only

**Gene names**	**Peptide ID**	**METTL 1/NLS-1**	**METTL 1/NLS-2**	**METTL 1/NLS-3**
RPL14	YVRQAW*QKADINTK	M1 only	5.29	3.08
ANXA8L2	AW*IEQEGVTVK	2.36	M1 only	M1 only
APOF	TGLC*GY*SAPDMRGLR	1.61	7.92	M1 only
ZNF362	HAKAYC*CSMCGR	M1 only	M1 only	1.54
	H*AKAYCCSMCGR	M1 only	M1 only	1.54
FRAS1	GEVRC*H*KQAC*LPLR	M1 only	M1 only	M1 only
	GEVRC*H*KQAC*LPLR	M1 only	M1 only	M1 only
	GEVRC*H*KQAC*LPLR	M1 only	M1 only	M1 only
HIPK1	TLNHQFVTMTH*LLDFPH*SN H*VK	M1 only	6.10	1.55
PBX4	EFTTH*VTNLLQEQSRMR	M1 only	M1 only	M1 only
VIM	SY*VTTSTR	M1 only	M1 only	M1 only
ACTG1;ACTB	GY*SFTTTAER	M1 only	M1 only	M1 only
	TTGIVMDSGDGVTHTVPIYEG Y*ALPHAILR	3.70	2.76	3.88
EIF4A1;EIF4A2	GY*DVIAQAQSGTGK	M1 only	5.65	M1 only
YBX1	NYQQNY*QNSESGEKNEGSES APEGQAQQR	M1 only	M1 only	M1 only
ACTA1;ACTC1; ACTG2;ACTA2	Y*PIEHGIITNWDDMEK	M1 only	M1 only	M1 only
EEF1A1;EEF1A1 P5;EEF1A2	EHALLAY*TLGVK	9.86	M1 only	3.31
TUBA1B;TUBA 4A;TUBA1A;TU BA3E;TUBA1C	VGINY*QPPTVVPGGDLAK	12.9	10.9	5.38
PABPC4	AHLTNQY* MQR	M1 only	M1 only	M1 only
CAPRIN1	DGY*QQNFK	M1 only	M1 only	M1 only
TFG	NRPPFGQGYTQPGPGY*R	M1 only	M1 only	M1 only

Table S3. Sequences of sgRNAs used for knocking out METTL1.

Description	Primer
sgRNA-1	5'-acgtgctcactccaacccca-3'
sgRNA-2	5'-ctatgtacaagaccggattc-3'

Figure S1. (A) Western blot analysis confirming comparable expression levels of METTL1-V5-APEX, EGFP-V5-APEX, and NLS-V5-APEX constructs. GAPDH and histone H3 served as loading controls for cytoplasmic and nuclear fractions, respectively. (B) Western blot analysis of METTL1 expression in Ctrl and *METTL1*$^{-/-}$ HEK293T cells. Whole cell lysate, cytosolic, and nuclear fractions were probed for METTL1. α-tubulin and GAPDH were used as cytosolic markers, while Lamin B1 and Histone H3 served as nuclear markers.

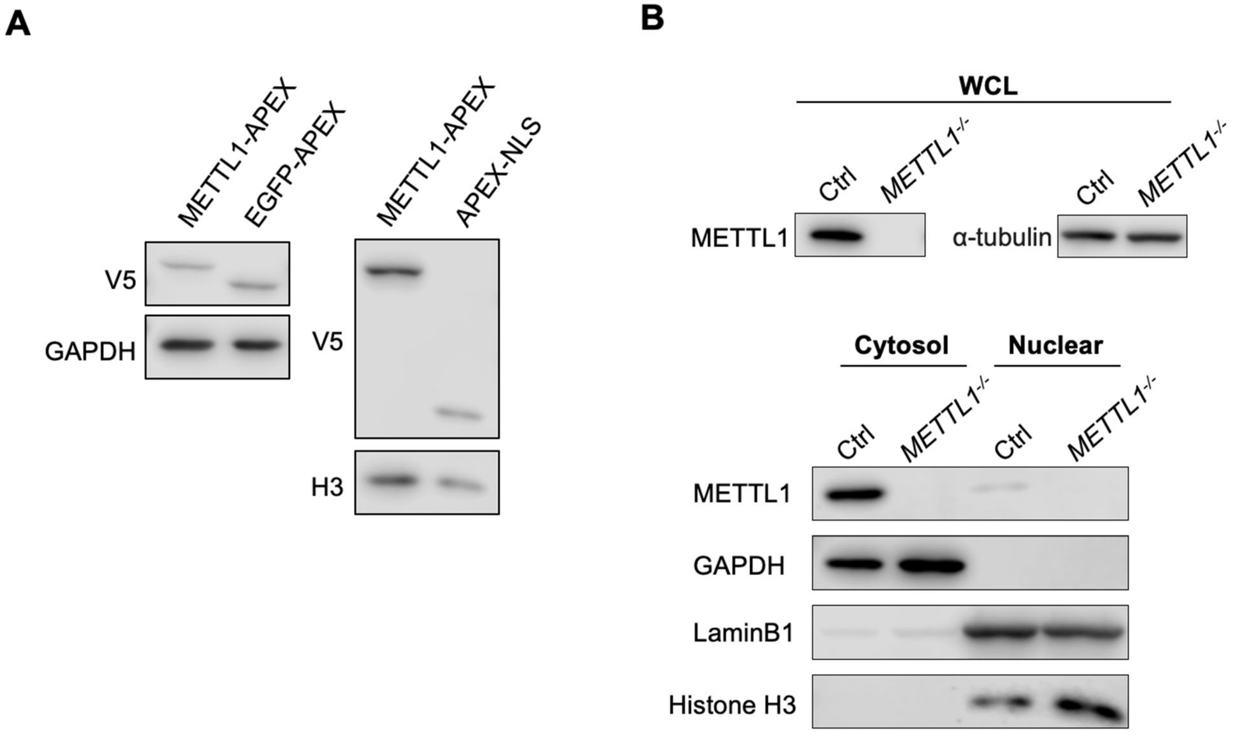

Figure S2. (A) STRING network analysis of the cytosolic proximity proteome of METTL1 identified by APEX2 labeling. Node size and color intensity correspond to the degree of connectivity (number of interactions), with larger and darker orange nodes representing proteins with higher degrees. (B) Gene Ontology (GO) analysis of proteins identified in the METTL1 proximal proteome.

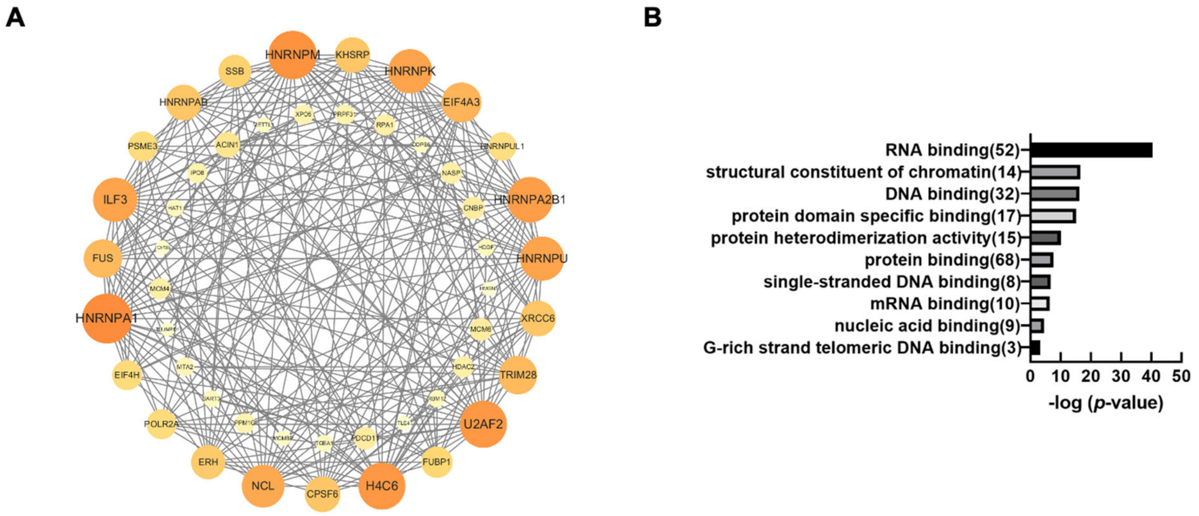

Figure S3. (A) Selected-ion chromatograms for monitoring the $[M + 3H]^{3+}$ ion (m/z 697.67) of a tryptic peptide of XPO5 (AGGFVVGY*TSSGNPIFR) in the digestion mixture of proximity proteomes of METTL1. (B) MS/MS of the $[M+3H]^{3+}$ ion of AGGFVVGY*TSSGNPIFR, where b and y ions are labeled in blue and red, respectively. (C) Alpha Fold-based structural prediction of putative interactions between METTL1 (green) and XPO5 (grey). Ribbon diagrams depicting the predicted protein folding, where thinner coils indicate regions with lower prediction confidence. Corresponding predicted aligned error (PAE) plots illustrate the confidence in inter- and intra-molecular residue positioning. Dark green regions represent higher prediction confidence, while lighter shades indicate increased positional uncertainty.

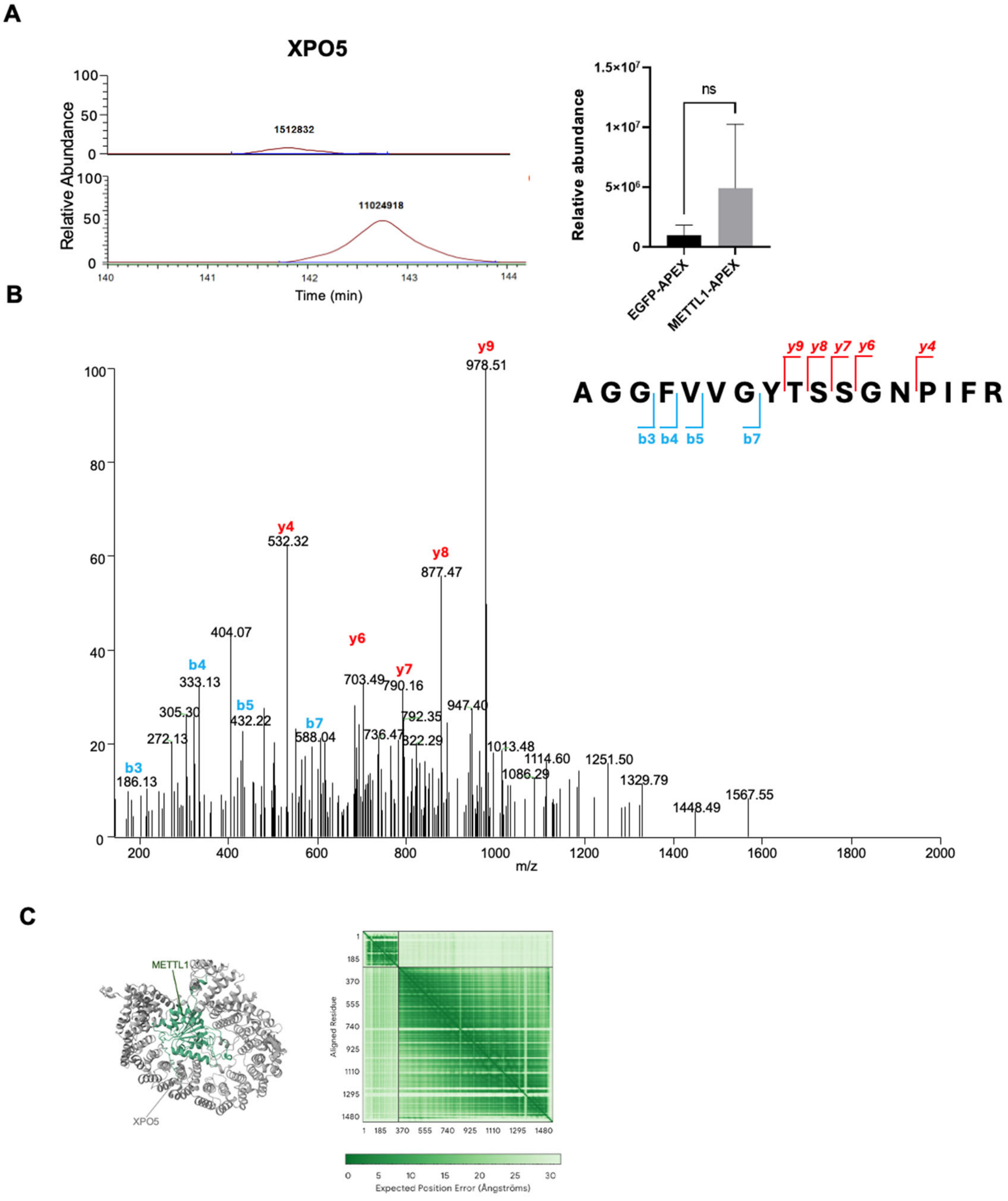

Figure S4. **(A)** Western blot confirming the similar expression level of wild-type METTL1 and its catalytically inactive mutant ($METTL1^{AFPA}$) in HEK293T cells, α-tubulin was used as the internal control.

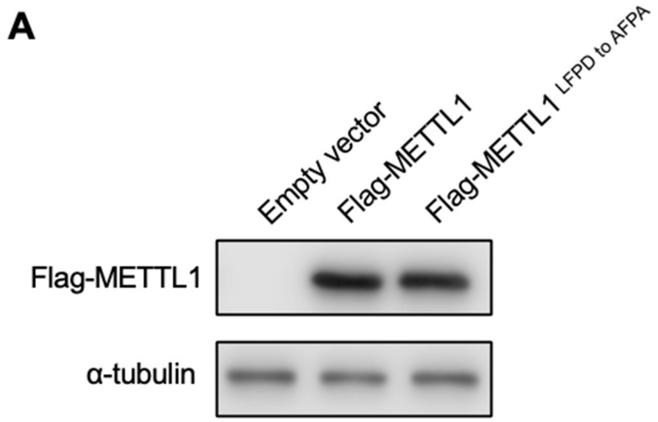

Supplement: gkag037_Supplemental_File [file gkag037_supplemental_file.pdf]
